# Supplementary material for: False versus True Statin Intolerance in Patients with Peripheral Artery Disease
Source: J Clin Med. 2022 Nov 8;11(22):6619. doi: 10.3390/jcm11226619 (PMC9695369; doi:10.3390/jcm11226619)
Supplement: Supplementary file 1 [file jcm-11-06619-s001.zip › jcm-1922842-supplementary.pdf]

Journal of
Clinical Medicine

MDPI

Supplementary Materials

Table S1. History of lipid lowering therapy at baseline.

Lipid lowering therapy	All PAD patients (n = 139)	uSI (n=106)	ppSI (n=33)	p-value
**Statin therapy at 1st contact, n (%)**	119 (86)	87 (82)	32 (97)	**0.04**
**Statin dose/d at 1st contact(mg +/- SD)**	57.8 ± 34.9	57.0 ± 32.9	58.7 ± 40.7	0.83
**Myopathy reported, n (%)**	89 (64)	61 (58)	28 (85)	**<0.001**
Typical myopathy, n (%)	27 (30)	4 (7)	23 (82)	**<0.0001**
Atypical myopathy, n (%)	62 (70)	57 (93)	5 (18)	
**Tried Statin > 1, n (%)**	82 (49)	50 (47)	29 (88)	**<0.0001**
Tried 2 Statins, n (%)	58 (73)	39 (78)	19 (66)	**0.04**
Tried 3 Statins, n (%)	15 (19)	8 (16)	7 (24)	**0.04**
Tried 4 Statins, n (%)	5 (7)	3 (6)	2 (7)	0.59
Tried 5 Statins, n (%)	1 (1)	0 (0)	1 (3)	0.24

History of lipid lowering therapy at baseline when patients were seen in the vascular outpatient clinic for the first time. Data are presented as absolute numbers/ percentages (n/N) or means ± standard deviation (SD). PAD: peripheral artery disease.

Suppl. Fig. 1 A

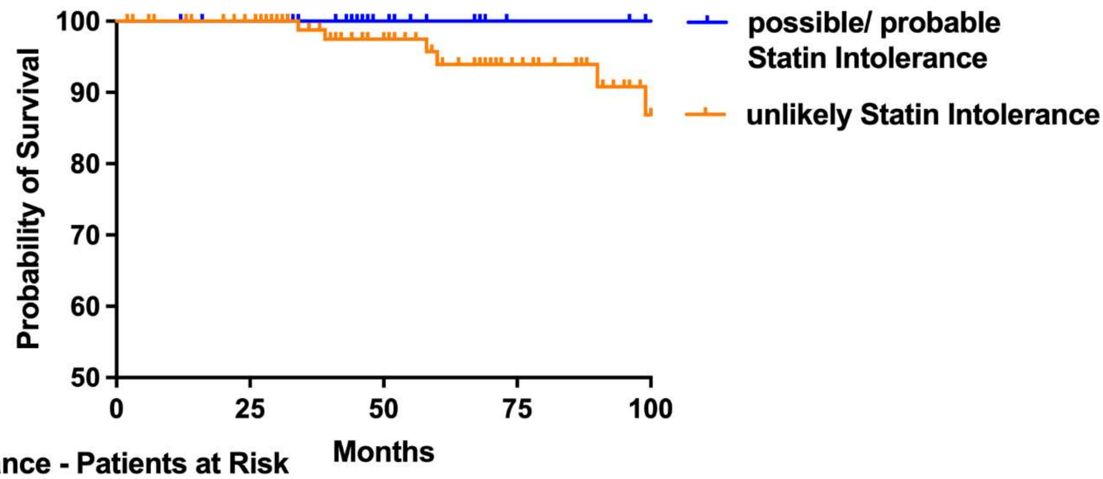

Statin Intolerance - Patients at Risk					
possible/probable:	33	32	18	8	5
unlikely:	106	95	61	41	23

Suppl. Fig. 1 B

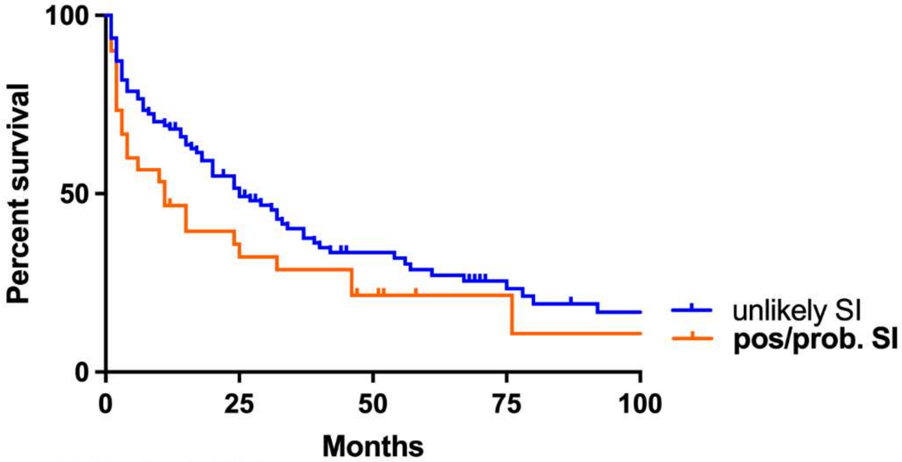

Statin Intolerance - Patients at Risk					
possible/probable:	33	10	5	3	2
unlikely:	106	44	22	12	8

Figure S1. Kaplan-Meier Diagrams of cardiovascular death (**A**) and cardiovascular events (**B**).
